# Supplementary material for: Selective consistency of recurrent neural networks induced by plasticity as a mechanism of unsupervised perceptual learning
Source: PLoS Comput Biol. 2024 Sep 3;20(9):e1012378. doi: 10.1371/journal.pcbi.1012378 (PMC11398647; doi:10.1371/journal.pcbi.1012378)
Supplement: S1 Table — (PDF) [file pcbi.1012378.s001.pdf]

Parameter	Variable	Value
Input dimension	N_{u}	1
Reservoir dimension	N_x	5×10^2
Reservoir connection density	d	10^{-1}
Internal noise level in the reservoir	ε	$10^{-1} \times N(0,1)$
Spectral radii	ρ	[0.1,0.9,1.0,1.1,1.2,1.3,1.4,1.5,1.6,1.7,1.8,1.9,2.0]
Output dimension	N_{y}	1
MBGD learning rate	η	10^{-1}
Hebbian learning rate	α	10^{-7}
Number of each stimulus type for a training dataset	-	5
Number of tests	-	200
Time points for transition	-	300
