# Supplementary material for: Selective consistency of recurrent neural networks induced by plasticity as a mechanism of unsupervised perceptual learning
Source: PLoS Comput Biol. 2024 Sep 3;20(9):e1012378. doi: 10.1371/journal.pcbi.1012378 (PMC11398647; doi:10.1371/journal.pcbi.1012378)
Supplement: S2 Table — (PDF) [file pcbi.1012378.s002.pdf]

Network	Spectral radius (ρ)												
Initial	0.1	0.9	1.0	1.1	1.2	1.3	1.4	1.5	1.6	1.7	1.8	1.9	2.0
non-Hebbian	0.1	0.9	1.0	1.1	1.2	1.3	1.4	1.5	1.6	1.7	1.8	1.9	2.0
**Hebbian**	**0.100**	**0.900**	**0.999**	**1.098**	**1.195**	**1.295**	**1.400**	**1.493**	**1.597**	**1.693**	**1.802**	**1.892**	**1.991**
