# Supplementary figures and images for: Selective consistency of recurrent neural networks induced by plasticity as a mechanism of unsupervised perceptual learning
Source: PLoS Comput Biol. 2024 Sep 3;20(9):e1012378. doi: 10.1371/journal.pcbi.1012378 (PMC11398647; doi:10.1371/journal.pcbi.1012378)

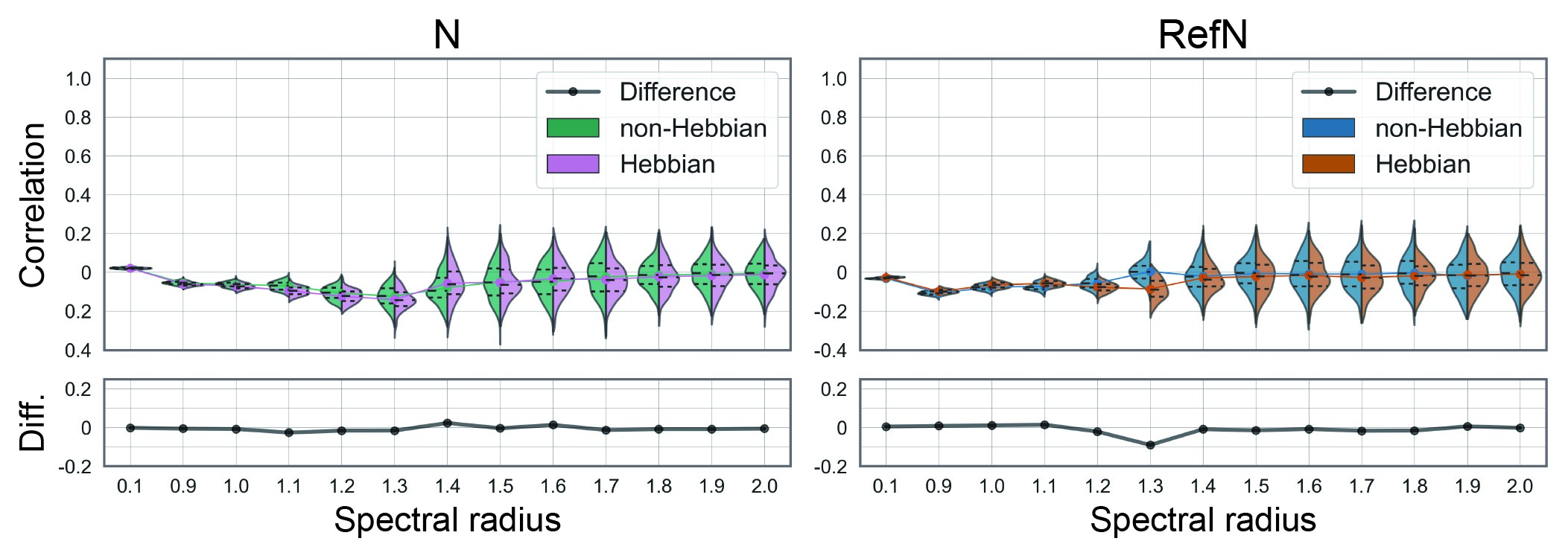

Supplement: S1 Fig — The evaluation of selective consistency for RefN (right panel) and N (left panel) stimuli. The figure style is the same as Fig 4A. The consistency was evaluated by the correlation between the first and second segment time series for each test run for repeated noise (N; left) and referenced repeated noise (RefN; right). The violin plots show probability density distributions and interquartile ranges of Hebbian (right side; magenta and brown) and non-Hebbian (left side; green and cyan) models, respectively. The colored line plots connect the mean values for each condition. The black lines in the bottom windows show the difference between Hebbian and non-Hebbian models. The horizontal axis represents the spectral radius of the evaluated networks. (TIF) [file pcbi.1012378.s003.tif]

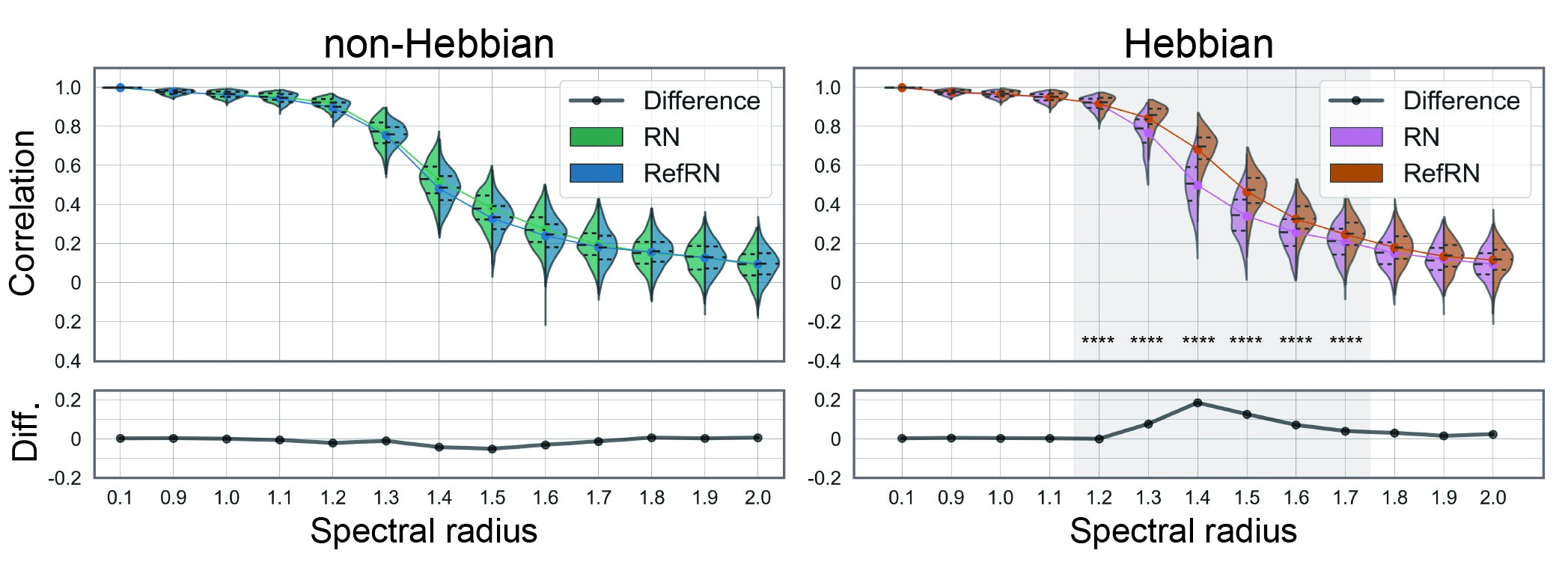

Supplement: S2 Fig — The evaluation of selective consistency for RN and RefRN of non-Hebbian (left panel) and Hebbian (right panel) networks. The figure style is the same as Figs 4A and S1. Colors for each condition are as follows: non-Hebbian RN; green, non-Hebbian RefRN; cyan, Hebbian RN; magenta, and Hebbian RefRN; brown. (TIF) [file pcbi.1012378.s004.tif]

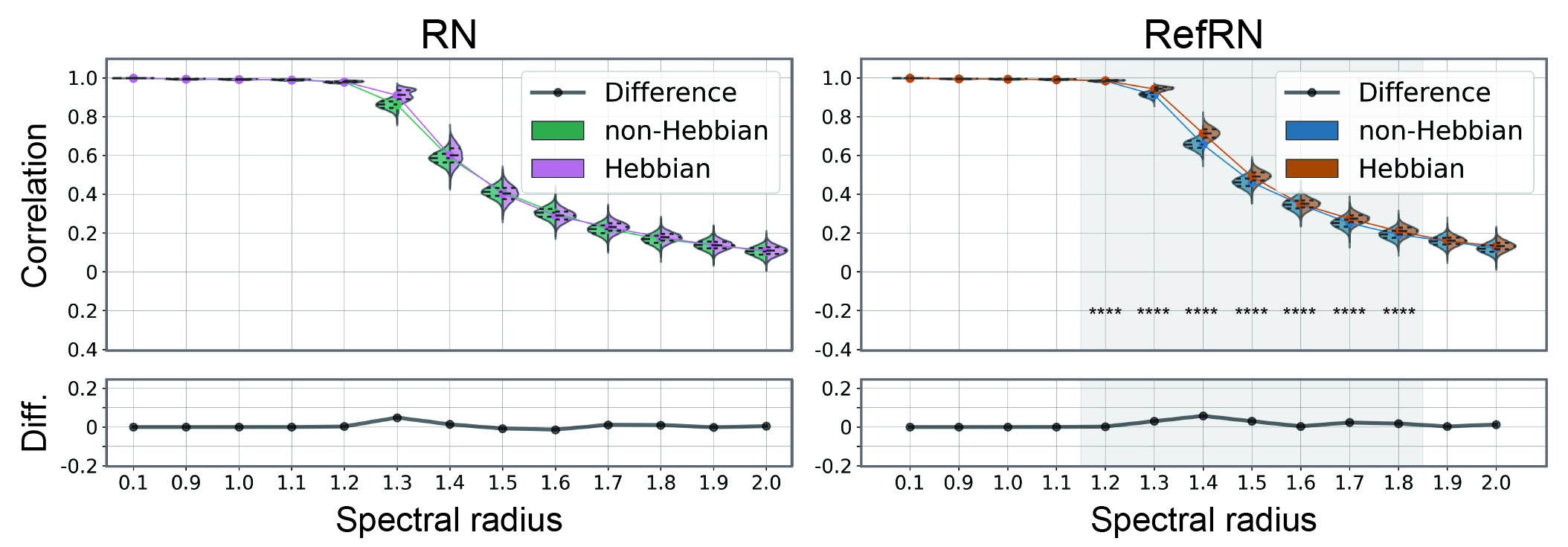

Supplement: S3 Fig — The inter-trial level selective consistency for RefRN (right panel) and RN (left panel). The consistency was evaluated by the mean of the correlation between all time series. The violin plots show probability density distributions and interquartile ranges of Hebbian (right side; magenta and brown) and non-Hebbian (left side; green and cyan) models, respectively (****; PR < 0.01%, p < 0.001). The colored line plots connect the mean values for each condition. The black lines in the bottom windows show the difference between Hebbian and non-Hebbian models. The horizontal axis represents the spectral radius of the evaluated networks. Weaker but significant differences between conditions can be seen in the same way as the inter-segment level comparison. (TIF) [file pcbi.1012378.s005.tif]
